# Synthesis, DFT Calculation, and Antimicrobial Studies of Novel Zn(II), Co(II), Cu(II), and Mn(II) Heteroleptic Complexes Containing Benzoylacetone and Dithiocarbamate

**DOI:** 10.1155/2015/789063

**Published:** 2015-11-22

**Authors:** Anthony C. Ekennia, Damian C. Onwudiwe, Lukman O. Olasunkanmi, Aderoju A. Osowole, Eno E. Ebenso

**Affiliations:** ^1^Department of Chemistry, Federal University Ndufu-Alike, Ikwo, PMB 1010, Abakaliki, Ebonyi, Nigeria; ^2^Material Science Innovation and Modelling (MaSIM) Research Focus Area, Faculty of Agriculture, Science and Technology, North-West University (Mafikeng Campus), Private Bag X2046, Mmabatho, South Africa; ^3^Department of Chemistry, School of Mathematical and Physical Sciences, Faculty of Agriculture, Science and Technology, North-West University (Mafikeng Campus), Private Bag X2046, Mmabatho 2735, South Africa; ^4^Department of Chemistry, Faculty of Science, Obafemi Awolowo University, Ile-Ife 220005, Nigeria; ^5^Inorganic Unit, Department of Chemistry, University of Ibadan, Ibadan, Nigeria

## Abstract

Heteroleptic complexes of zinc(II), copper(II), manganese(II), and cobalt(II) of the types [MLL′(H_2_O)_2_]·*n*H_2_O and [MLL′]·*n*H_2_O have been synthesized using sodium *N*-methyl-*N*-phenyldithiocarbamate (L) and benzoylacetone (L′). The metal complexes were characterized by elemental analysis, electrical conductance, magnetic susceptibility, infrared (IR), and UV-visible spectroscopic studies. The electrical conductance measurements revealed the nonelectrolytic nature of the synthesized complexes. The results of the elemental analyses, magnetic susceptibility measurements, and electronic spectra inferred that the Zn(II) complex adopted a four-coordinate geometry while the Co(II), Cu(II), and Mn(II) complexes assumed octahedral geometries. The IR spectra showed that the metal ions coordinated with the ligands via the S- and O-donor atoms. The geometry, electronic, and thermodynamic parameters of the complexes were obtained from density functional theory (DFT) calculations. The spin density distributions, relative strength of H–bonds, and thermodynamic parameters revealed that the order of stability of the metal complexes is Mn < Co < Cu > Zn. The agar diffusion methods were used to study the antimicrobial activity of the complexes against two Gram positive bacteria (*S. aureus* and *S. pneumoniae*), one Gram negative bacterium (*E. coli*), and two fungi organisms (*A. niger* and *A. candida*) and the complexes showed a broad spectrum of activities against the microbes.

## 1. Introduction

Medicinal inorganic chemistry has generated significant interest in the design of metal complexes as potential diagnostic and therapeutic agents. There are several metal complexes that are already in use for these purposes and this has encouraged further research on new metallodrugs such as metal-mediated antibiotics and anticancer and antiviral compounds [1]. The coordination chemistry of transition metal complexes with more than one type of ligands is of current interest because they serve as models for biochemical reactions [2]. Also, they provide new materials with useful properties such as magnetic exchange [3, 4], electrical conductivity [5], photoluminescence [6], and nonlinear optical property [7]. Mixed ligand complexes play important roles in biological processes like activation of enzymes by metals [8, 9] and storage and transport of active substances through membranes [10]. They have also been reported as being biologically active against pathogenic microorganisms [11–13]. Metal complexes containing different ligands with oxygen and sulfur donor atoms have been reported in the literature, and among these highlights are the dithiocarbamates and *β*-diketones mixed ligand complexes [14–16].

**Figure 1:**
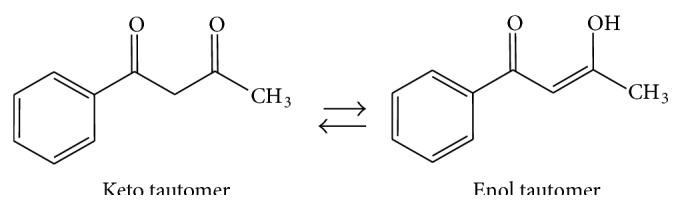
Keto-enol tautomerism of benzoylacetone (*β*-diketone).

The chemistry of *β*-diketones (R_1_COCHR_3_COR_2_) has drawn a lot of interest in inorganic chemistry due to their varied coordination modes and their ability to exhibit keto-enol tautomerism (Figure 1) [17, 18]. Their tautomeric forms are as a result of prototropic shift of the hydrogen atom of the CHR_3_ group which is activated by the adjacent C=O groups, leading to conjugate system [19]. Substitution has a pronounced effect on tautomeric forms as the presence of electron withdrawing groups or phenyl at the R_1_ and/or R_2_ position(s) increases enolisation while electron releasing substituents like alkyl and methoxy increased the keto tautomers [20–22]. Spectroscopic characterization (mostly infrared spectra) of various *β*-diketones has shown that these tautomers exist in equilibrium with each other [23]. *β*-Diketones metal complexes have been reported to possess several biological properties [24, 25]. Similarly, dithiocarbamates and their metal complexes have been widely studied because of their wide biological, industrial, agricultural, and chemical applications [26–34]. They have been used as nitrogen-oxygen trapping agents [26], chelating agents of heavy metals [27–29], vulcanizers, fungicides, lubricants, and catalysts [30]. They have also been used in medicine since the dithiocarbamate moiety has been found in a variety of biologically active molecules [30–35].

Herein, we report the synthesis, antimicrobial properties, and the DFT studies of some novel heteroleptic complexes involving* N*-methyl-*N*-phenyldithiocarbamate and benzoylacetone. The aim is to explore their potency as novel bactericidal and fungicidal agents and also study the electronic and thermodynamic properties of the heteroleptic systems generated due to the presence of the two different ligand molecules in a compound.

## 2. Experimental

### 2.1. Materials and Methods

Copper(II) sulphate pentahydrate, cobalt(II) sulphate heptahydrate, zinc(II) sulphate heptahydrate, manganese(II) nitrate hexahydrate, carbon disulfide, benzoylacetone, and* N*-methyl aniline (Aldrich) were used as received. Methanol and diethyl ether (Ace Chemicals) were used directly.

Elemental analyses (C, H, N, and S) were performed on an Elementar, Vario EL Cube, setup for CHNS analysis. The measurements of the room temperature magnetic susceptibilities were performed using a Johnson Matthey magnetic susceptibility balance, and the diamagnetic corrections were calculated using Pascal's constant [36]. The molar conductivity of the complexes was conducted using a MC-1 conductivity meter with a cell constant of 1.0 measured at 25°C [37]. Electronic absorption spectra of the solutions were recorded on a Perkin Elmer Lambda 40 UV-Vis spectrometer. FTIR spectra (400–4000 cm^−1^ region) were recorded on a Bruker alpha-P FTIR spectrometer.

### 2.2. Synthesis

#### 2.2.1. Preparation of Metal Complexes of Benzoylacetone and* N*-Methyl-*N*-phenyldithiocarbamate

**Figure 2:**
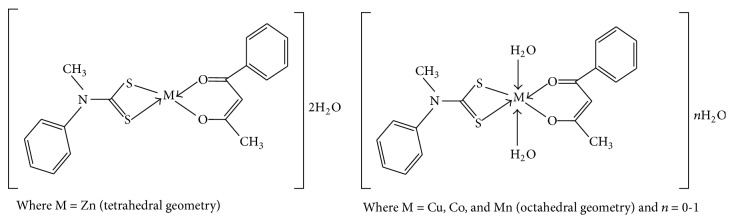
Proposed geometry of the mixed ligand complexes.

**Figure 3:**
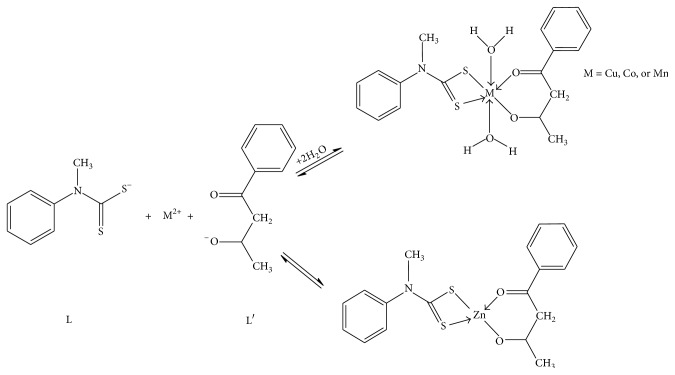
Schematic presentation of the synthesis of the complexes.

Sodium* N*-methyl-*N*-phenyldithiocarbamate was synthesized according to a published procedure [38]. Equimolar concentration of sodium* N*-methyl-*N*-phenyldithiocarbamate (2.4 mmol, 0.5 g) and benzoylacetone (2.4 mmol, 0.39 g) was dissolved in ethanol and the solution was added to 2.4 mmol of the respective metal salt (CoSO_4_·7H_2_O, CuSO_4_·5H_2_O, ZnSO_4_·7H_2_O, or Mn(NO_3_)_2_·6H_2_O). Triethylamine (0.3 mL) was added in drops to the reaction mixture. The resultant mixture was stirred at room temperature for 3 h. The metal complexes were obtained as precipitates which were filtered under vacuum and stored under silica gel. The proposed structures and the schematic presentation of the synthesis of the mixed complexes are presented in Figures 2 and 3.

Cobalt complex; (CoLL′): [CoC_18_H_21_NS_2_O_4_] (Yield: 0.30 g, 86%), Elemental analysis, Anal: C, 49.31; H, 4.84; N, 3.20; S, 14.62. Found: C, 49.28; H, 4.82; N, 3.17; S, 14.58. FTIR (v/cm^−1^): 3435b, 3007m, 2989m, 2875m 1605s, 1585s, 1512s, 1462s, 1432s, 1256m, 1243m, 913s, 432m. UV-Vis: 14930, 15580, 24940, 25010, and 28570 cm^−1^. Magnetic moment: 4.9 BM; Conductance (*Ω* ^−1^ cm^2^ mol^−1^): 59.5.

Copper complex; (CuLL′)·H_2_O: [CuC_18_H_21_NS_2_O_4_]·H_2_O (Yield: 0.32 g, 92%), Elemental analysis, Anal: C, 46.89; H, 5.04; N, 3.04; S, 13.91. Found: C, 46.84; H, 5.05; N, 3.03; S, 13.88. FTIR (v/cm^−1^): 3478b, 3013m, 2945m, 2823m 1603s, 1587s, 1542s, 1452s, 1426s, 1253m, 1247m, 907s, 419m. UV-Vis: 14990, 25190, 29240, and 40320 cm^−1^. Magnetic moment: 2.00 BM; Conductance (*Ω* ^−1^ cm^2^ mol^−1^): 5.47.

Zinc complex; (ZnLL′)·2H_2_O: [ZnC_18_H_17_NS_2_O_2_]·2H_2_O (Yield: 0.30 g, 86%), Elemental analysis, Anal: C, 48.59; H, 4.77; N, 3.15; S, 14.41. Found: C, 48.54; H, 4.73; N, 3.12; S, 14.28. FTIR (v/cm^−1^): 3480b, 3001m, 2921m, 2811m 1600s, 1580s, 1544s, 1433s, 1420s, 1263m, 1257m, 933s, 456m. UV-Vis: 24940, 29500, 33000, and 40320 cm^−1^. Magnetic moment: 0.12 BM; Conductance (*Ω* ^−1^ cm^2^ mol^−1^): 9.32.

Manganese complex; (MnLL′): [MnC_18_H_21_NS_2_O_4_] (Yield: 0.32 g, 92%), Elemental analysis, Anal: C, 49.76; H, 4.88; N, 3.23; S, 14.76. Found: C, 49.70; H, 4.83; N, 3.21; S, 14.73. FTIR (v/cm^−1^): 3503b, 3008m, 2934m, 2851m 1612s, 1595s, 1552s, 1423s, 1411s, 1234m, 1212m, 907s, 448m. UV-Vis: 11570, 15580, 24940, 29670, and 41150 cm^−1^. Magnetic moment: 5.82 BM; Conductance (*Ω* ^−1^ cm^2^ mol^−1^): 43.3.

## 3. DFT Computational Studies

Geometry optimization and frequency calculations were carried out on the two ligands, that is,* N*-methyl-*N*-phenyldithiocarbamate (L) and benzoylacetone (L′), and their Zn(II), Cu(II), Co(II), and Mn(II) complexes. All the optimized structures were confirmed to correspond to the most stable ground state conformers by the absence of imaginary frequency in the force constant calculations. Since the ligands are expected to dissociate into their corresponding anions in solution, the optimized structures of the ligands are those of their singly charged anions. The density functional theory (DFT) method involving the Becke 3-parameter exchange functional together with the Lee-Yang-Parr correlation functional (B3LYP) [39, 40] was used for all the calculations. The B3LYP functional has been successfully used in some previous works for geometry optimization of transition metal complexes [41–44]. It has proven sufficient to produce acceptable geometry and spectroscopic parameters comparable to experimental crystallographic data for some transition metal complexes at moderate computational cost [44]. The 6-31+G(d,p) basis set was used for C, H, N, O, and S atoms, while the metal ions were described by the LANL2DZ relativistic pseudopotential. The LANL2DZ relativistic pseudopotential has been found reliable for quantum chemical studies on transition metal complexes [43, 45–50]. It is a “double” quality basis set which uses the Duning D95 V basis set on the first-row atoms and Los Alamos ECP plus DZ on Na-Bi [42, 51–54]. It has been reported to be computationally efficient and suitable for a variety of transition metal complexes [42, 55–57]. The placement of the ECP on transition metal ions via the use of the LANL2DZ basis set has been found to yield results at similar level of accuracy to the all-electron basis set, such as DZVP [42, 58]. DFT computational model similar to the one used in the present work has been previously employed by Gorelsky et al. for theoretical description of some metal complexes of sulphur containing chelating resin [44].

All the ligands and metal complexes were modeled with Gaussview 5.0 software. Based on the results obtained from the magnetic moment experiments, a four-coordinate system was adopted for the ZnLL′, while CuLL′, CoLL′, and MnLL′ were modeled as six-coordinate systems each with two molecules of water as additional ligands. Gas phase geometry optimizations were carried out without symmetry constraint by using the Gaussian 09W software [59].

Geometry and electronic and thermodynamic parameters were obtained from the optimized geometries. The frontier molecular orbital (FMO) energies, the energy of the highest occupied molecular orbital, *E*_HOMO_, and the energy of the lowest unoccupied molecular orbitals, *E*_LUMO_, of the studied metal complexes are reported. The binding energy, BE, was calculated for each of the metal complexes as the energy required to disassemble the metal complex into its constituent ligands and metal ion, equivalent to the energy difference for the reaction equation shown in Figure 3.

According to the equation shown in Figure 3, BE was calculated as

(1) $$\begin{array}{c} \text{BE}=E_{\text{(M-complex)}}-\left(\;E_{\left(L\right)}+E_{\left(L^{\prime }\right)}+nE_{\left(H_{2}O\right)}+E_{\left(M^{2+}\right)}\;\right), \end{array}$$

where *E*_(M-complex)_ is the energy of the metal complex (ZnLL′, MnLL′, CoLL′, or CuLL′), *E*_(M^2+^)_ is the energy of the metal ion (Zn(II), Cu(II), Co(II), or Mn(II)), and *E*_(L)_ and *E*_(L′)_ are the energies of the ligands L and L′, respectively. The constant *n* = 0 for ZnLL′and equals 2 for other complexes. Other thermodynamic parameters such as the change in enthalpy (Δ*H*), entropy (Δ*S*), and Gibb's free energy (Δ*G*) of complexation were calculated according to the following equation:

(2) ΔH=HM-complex−HL+HL′+nHH2O+HM2+,ΔS=SM-complex−SL+SL′+nSH2O+SM2+,ΔG=GM-complex−GL+GL′+nGH2O+GM2+,

where *H*_*i*_, *S*_*i*_, and *G*_*i*_ are the enthalpy, entropy, and free energy, respectively, of the corresponding species in the equation shown in Figure 3.

## 4. Results and Discussion

### 4.1. Electronic Spectra and Magnetic Moment

The absorption spectroscopy of the complexes was carried out as solid reflectance and recorded in wave numbers (cm^−1^). The ultraviolet region of the electronic spectrum of the complexes was characterized by n → *π* ^*∗*^ and *π* → *π* ^*∗*^ transitions of the ligands at 28570, 29240, 29500, 29670, 33000, 40000, 40320, and 41150 cm^−1^ [38].

The visible spectrum of copper(II) complexes is usually complicated due to the unsymmetrical band which arise from the Jahn-Teller distortions resulting in a number of overlapping bands. Jahn-Teller distortion in Cu(II) complexes is a consequence of the uneven distribution of electrons in the e_g_ set of the 3d orbitals [60]. Copper(II) complexes of a regular tetrahedral geometry usually have, in the visible region of the electronic spectrum, a single broad band below 10000 cm^−1^ of approximately 10^2^ molar intensity which is ascribed to ^2^T_2_ → ^2^E transition, while regular octahedral copper(II) complexes have a single broad band above 10000 cm^−1^, with molar absorptivities of 10^3^ M^−1^ cm^−1^, which is ascribed to ^2^E → ^2^T_2_ transition. Copper(II) complexes with a square-planar geometry, usually in its visible region of the electronic spectrum, show two bands between 15000 and 20000 cm^−1^ with molar absorptivities of 10^2^ M^−1^ cm^−1^ [60, 61]. The copper complex showed a single absorption band at 14990 cm^−1^ and is ascribed to the ^2^E → ^2^T_2_ transitions of an octahedral geometry. For copper complexes, the magnetic moment values are usually not used for prediction of the geometry but could give information on the number of metal centers involved in the complex. A moment of 1.9–2.2 BM is usually observed for mononuclear copper(II) complexes, regardless of stereochemistry, expectedly higher than the spin-only moment due to orbital contribution and spin-orbit coupling. A higher value may be seen in dinuclear copper complexes [62]. The copper(II) complex displayed a magnetic moment of 2.0 BM indicating its mononuclear nature.

Cobalt complex in a tetrahedral environment gives rise to three bands in the visible region of the electronic spectra that are ascribed to ^4^A_2_ → ^4^T_2_(F) (*ν*_1_), ^4^A_2_ → ^4^T_1_(F) (*ν*_2_), and ^4^A_2_ → ^4^T_1_(P)  (*ν*_3_) transitions. The (*ν*_1_) transition is usually not seen because it falls within the infrared region; (*ν*_2_) transition usually appears in the near infrared region, while (*ν*_3_) transition occurs in the visible region. The magnetic moment for cobalt(II) complex in a tetrahedral environment is within 4.20–4.60 BM but may be higher for stronger field ligands. Octahedral cobalt(II) complexes typically have three absorption bands in the visible region that are ascribed to ^4^T_1_g(F) → ^4^T_2_g(F)  (*ν*_1_), ^4^T_1_g(F) → ^4^A_2_g(F)  (*ν*_2_), and ^4^T_1_g(F) → ^4^T_1_g(P) (*ν*_3_). This geometry could further be corroborated with magnetic moment value which falls in the range 4.7–5.2 BM [63]. The reflectance spectra of the cobalt complex showed three absorption bands at 14930, 15580, and 24940 cm^−1^ ascribed to ^4^T_1_g(F) → ^4^T_2_g(F) (*ν*_1_), ^4^T_1_g(F) → ^4^A_2_g(F), (*ν*_2_), and ^4^T_1_g(F) → ^4^T_1_g(P), (*ν*_3_), respectively, of an octahedral geometry [36, 64]. The magnetic moment for the complex is 4.98 BM.

Zn(II) complexes usually do not have d-d absorption bands in the visible region due to the presence of completely filled 3d orbitals but display metal → ligand transitions. Zinc(II) metal complexes are diamagnetic in nature with a magnetic moment below 0 BM and mostly adopt a four-coordinate tetrahedral geometry [65]. The zinc complex showed a single charge transfer absorption band at 24940 cm^−1^, in the visible region and a magnetic moment of 0.12 BM.

The Mn(II) complexes are characterized by weak spin forbidden transitions. This is due to the presence of a ^6^S ground term and a ^4^G upper term. An octahedral Mn(II) complex is usually characterized by three weak absorption bands due to ^6^A_1_g → ^4^T_2_g (G), ^6^A_1_g → ^4^E_g_(G), and ^6^A_1_g → ^4^T_1_g transitions [66]. The manganese complex showed three weak absorption bands at 11570, 15580, and 24940 cm^−1^ typical of 6-coordinate octahedral geometry and are assigned to ^6^A_1_g → ^4^T_1_g, ^6^A_1_g → ^4^T_2_g(G), and ^6^A_1_g → ^4^E_g_(G) transitions, respectively. The effective magnetic moment of Mn(II) complexes is expected to be close to the spin-only value of 5.90 BM. Since the ground term is ^6^A_1_g, there is no orbital contribution. Consequently, an observed moment of 5.67 BM for this complex indicates that it is high spin and complementary of octahedral geometry [67].

### 4.2. Infrared Spectra

The infrared spectra of the complexes gave bands between 400 and 4000 cm^−1^ from which information about the mode of coordination of the two ligands to the metal ions could be deduced. Bands due to O–H stretching of the water of crystallization in the complexes appeared around 3503–3435 cm^−1^. The hydrogen stretching bands due to the aromatic phenyl ring, *ν*(Ar–H), occurred as medium bands between 3013 and 3001 cm^−1^ in the complexes. The C–H stretching bands for the alkyl groups of the dithiocarbamate moiety were observed around 2989–2811 cm^−1^. The C=O stretching bands of the coordinated carbonyl group of the benzoylacetone in the enol form appeared as sharp bands between 1612 and 1600 cm^−1^, while the C–O stretching vibration of the benzoylacetone and the *ν*(C_2_–N) frequency of the dithiocarbamate ligand appeared as sharp band around 1263–1212 cm^−1^. The *ν*(C=N) stretching bands of the dithiocarbamate moiety occurred as sharp bands in the range 1462–1411 cm^−1^. The *ν*(C=S) frequency appeared as single bands around 933–907 in all the complexes and indicates a symmetrical bonding of the sulfur atoms of the dithiocarbamate ligand to the central metal ion. Stretching bands of the *ν*(M–O) from the benzoylacetone moiety can be seen around 456–419 cm^−1^. The *ν*(M–S) stretching bands for dithiocarbamate complexes usually fall below 400 cm^−1^ and thus could not be observed due to the spectral range of the measurements.

### 4.3. Conductivity Measurements

The metal complexes have molar conductivities (Λm) of 5.47–59.5 ohm^−1^ cm^2^ mol^−1^ in DMSO and are therefore nonelectrolytes since a value above 60 ohm^−1^ cm^2^ mol^−1^ is expected for 1 : 1 electrolyte [68]. Higher values of molar conductivities observed in the Co(II) and Mn(II) complexes compared to the Zn(II) and Cu(II) complexes may be due to the presence and absence of solvolysis in the complexes rather than ionic dissociation. Solvolysis is a special type of nucleophilic substitution (S_N_1) or elimination where the nucleophile is a solvent molecule. DMSO which was used for conductivity measurement is a coordinating solvent and capable of causing solvolysis [69].

### 4.4. Quantum Chemical Studies

**Figure 4:**
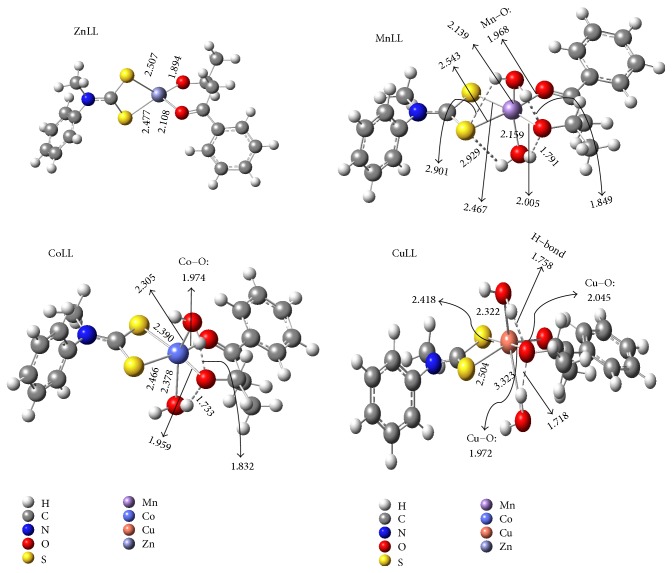
Optimized structures ZnLL′, MnLL′, CoLL′, and CuLL′ at B3LYP/6-31+G(d,p). Selected bond lengths (Å) that are relevant to the results and discussion are labeled.

The gas phase optimized structures of the studied mixed ligand complexes are shown in Figure 4. The selected bond lengths that are salient to the results and discussion are listed in Figure 4. As shown in the equation in Figure 3, there are two different types of M–S and M–O bonds in the metal complexes, apart from the M–OH_2_ bonds, which are only found in MnLL′, CoLL′, and CuLL′. For the purpose of discussion of results, the two different M–S and M–O bonds with which the central metal ion in each case binds with the ligands are designated as M–S_sp^2^_ and M–S_sp^3^_ and M–O_sp^2^_ and M–O_sp^3^_. The results in Figure 4 show that the trend of the M–O_sp^2^_ bond lengths in the studied complexes is Zn–O_sp^2^_ > Cu–O_sp^2^_ > Co–O_sp^2^_ > Mn–O_sp^2^_, while the trend of the M–O_sp^3^_ bond lengths is Mn–O_sp^3^_ > Cu–O_sp^3^_ > Co–O_sp^3^_ > Zn–O_sp^3^_. The trend of the M–O_sp^2^_ bond lengths is the direct opposite of the order of the atomic radius of the central metal atoms, which implies that the M–O_sp^2^_ bond lengths for the studied metal complexes do not have direct relationship with the atomic radius of the central metal. However, the shortest M–O_sp^2^_ bond length observed for the MnLL′ may be as a result of the presence of two vacant 3d orbitals in the singlet electronic configurations of Mn^2+^ ion, which may inform optimum interactions with the pi-electrons of the sp^2^ oxygen in the ligand.

The trend of the M–O_sp^3^_ bond lengths for the six-coordinate complexes is in line with the trend of the covalent radii of the central metal atoms. Meanwhile, the shortest M–O_sp^3^_ bond length observed for the ZnLL′ may be due to the higher percentage of s and p orbitals in the tetrahedral configurations (sp^3^) of the Zn^2+^ complex compared to the octahedral configurations in the other three complexes. This may allow for a higher degree of orbital interactions with the sp^3^ O atom. The trends of the M–S bond lengths however are not the same as the M–O bond lengths. The Co–S_sp^2^_ is the shortest, while the Cu–S_sp^3^_ is the shortest of the M–S_sp^2^_ and M–S_sp^3^_ bonds, respectively. Another important observation in Figure 4 is the strength of the hydrogen bonds, X- - -H (where -X = -S_sp^3^_, or -O_sp^3^_, and H is the H atom of the H_2_O molecules) with the decreasing strength in the order CuLL′ > CoLL′ > MnLL′ for the O_sp^3^_- - -H bonds. Only the MnLL′ shows a satisfactory evidence of S_sp^3^_- - -H bonds with bond lengths slightly above 2.9 Å. The S_sp^3^_- - -H bonds in other complexes are longer than this value and therefore cannot be considered as to exhibit significant H–bond characters. The two M–OH_2_ bond lengths in MnLL′, CoLL′, and CuLL′ follow the order of increasing atomic radii of the metal ions differing by 0.02 Å in MnLL′, 0.073 Å in CoLL′, and 1.001 Å in CuLL′. This reveals that one of the H_2_O molecules in the CuLL′ is displaced farther away from the central Cu(II), which may be due to the Jahn-Teller distortion usually observed in Cu(II) complexes. The M–O and M–S bond lengths observed for the studied metal complexes are in good agreement with what have been reported in the literature for similar bonds [70, 71].

**Figure 5:**
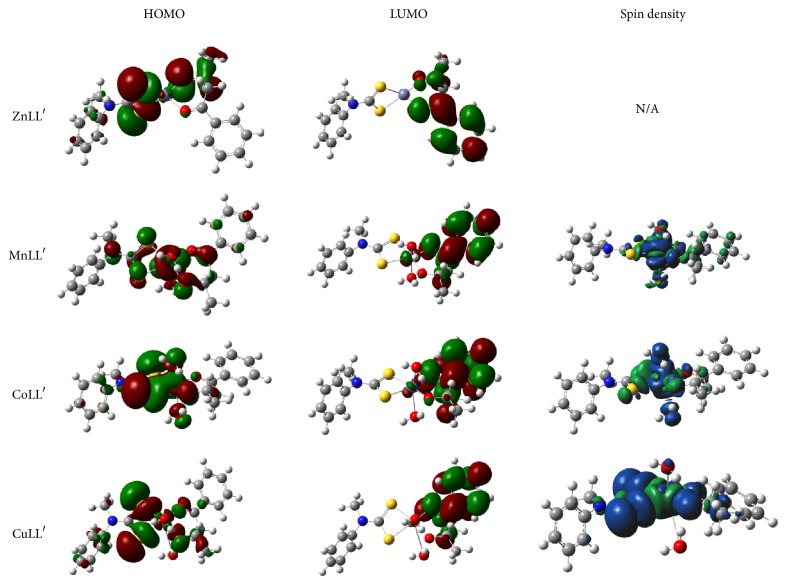
The graphical images of the HOMO, LUMO, and spin density electron distributions in ZnLL′, MnLL′, CoLL′, and CuLL′ at B3LYP/6-31+G(d,p)/LANL2DZ.

The graphical surfaces of the electron density distributions of the HOMO, LUMO, and spin delocalization in the studied metal complexes are shown in Figure 5. The HOMO of the ZnLL′ is essentially localized around the central Zn(II) ion and the neighboring coordination sites involving the atoms in the dithiocarbamate and alkoxyl groups of L and L′ ligands, respectively. Similar observations were made for the HOMO electron density distributions in MnLL′, CoLL′, and CuLL′, except that the O atoms of the water molecules also take part in the HOMO electron distributions. In all the studied metal complexes, the aromatic rings of the ligands do not make significant contributions to the HOMO. These electron density distributions of the HOMO surfaces suggest that the possible interactions of the studied metal complexes with an electrophilic agent will occur mainly around the central metal ions and the coordination sites and not around the aromatic rings. The electronegative S and O atoms that are directly bonded with the metal ions are capable of pulling electrons away from their respective adjacent aromatic rings, thereby decreasing the HOMO density around the rings. For all the studied metal complexes, the LUMO is essentially delocalized on the benzoylacetone ligand moiety. This implies that the most susceptible sites on the studied complexes for favourable interactions with an electron-rich species are located on the benzoylacetone unit.

The spin density distributions for the studied metal complexes are shown in Figure 5. For the complexes in which the central metal ion has “*n*” unpaired electrons in the valence orbitals, the molecular orbitals are said to be characterized with these *n* unpaired electrons such that a total spin density of +*n* should be expected for the singly occupied molecular orbitals (SOMOs). It is expected that a large percentage of the spins are credited to the d-orbitals of the metal, since they provide the major contributions to the SOMOs. A fraction of the spin density is often delocalized to the ligand atoms and the spin population at the metal is usually less than the number of unpaired electrons in the valence atomic orbitals of the metal. In this regard, the difference between the number of unpaired electrons and the total spin density at the metal atom can be employed as a measure of the degree of covalent character of the metal–ligand bonds [72, 73]. Therefore, spin density distribution could be a reliable parameter for predicting the relative strength of metal–ligand covalent bonds.

The higher the spin density extended to a ligand atom, the stronger the covalent bond between that atom and the central metal ion containing the unpaired electron(s). With the spin density distribution analyses, a nonbias comparative strength of similar metal–ligand covalent bonds in a family of complexes can be deduced irrespective of the atomic or covalent radii of the metal ions which can bring about a relatively longer or shorter bond length that may not correlate directly with the relative strength of the bonds.

Since Zn(II) is a d^10^ system in which all the electrons are paired, no residual spin is expected and, thus, the results obtained for the ZnLL′ showed no spin density. The results in Figure 5 reveal a clear localization of the spin density at the central metal ions in MnLL′, CoLL′, and CuLL′, which is in agreement with the presence of unpaired electron in the respective d-orbitals of the metal ions. The spin densities are also extended to the S and O atoms of the organic ligands and water molecules bonded to the central metal ions. The involvement of the ligand atoms that are around the coordination sites in the spin density distributions is more pronounced for CuLL′ than CoLL′ and MnLL′, with the Mn(II) ion showing the least delocalization of the unpaired electron spin onto the ligand molecular orbitals. The percentage contributions of some selected atoms to the spin density distributions were calculated as the ratio of the square of the Mulliken atomic spin density for an atom to the sum of the square of Mulliken spin density for all atoms and the results are presented in Table 1. The results showed that the percentage spin density on the central metal ions is in the order Mn(II) > Co(II) > Cu(II), which may be due to the presence of 2, 1, and 0 empty d-orbital(s) in Mn(II), Co(II), and Cu(II), respectively. In other words, vacant d-orbitals in the electronic configurations of the metal ion may serve as the hosts for the delocalized spin density and reduce the percentage of the spin density extended to the adjacent atoms. It can be inferred from the results in Table 1 that the d-orbital that hosts the unpaired electron in CuLL′ complex mixes better with the atomic orbitals of the ligands than the d-orbitals hosting the unpaired electrons in CoLL′ and MnLL′ complexes. The results suggest further that the decreasing strength of the covalent bonds between the central metal ion in each case and the ligand atoms involved in coordination with the metal ion is CuLL′ > CoLL′ > MnLL′.

Some electronic and thermodynamic parameters were calculated for the metal complexes and the results are listed in Table 2. The frontier molecular orbital energy parameters such as the *E*_HOMO_, *E*_LUMO_, and Δ*E*_LUMO-HOMO_ are often used as reactivity or stability indices. A high value of *E*_HOMO_ implies better tendency of a molecule to donate its most loosely bound electron to the appropriate orbitals of an acceptor molecule. The decreasing order of *E*_HOMO_ of the studied metal complexes is MnLL′ > CoLL′ > CuLL′ > ZnLL′ which implies that the Zn^2+^ complex has the highest tendency to donate its most energetic electron to a suitable orbital of an acceptor molecule. The *E*_LUMO_ is a measure of the tendency of a molecule to accept electrons from the appropriate orbital of a donor species. The lower the *E*_LUMO_ the better the chance of electron acceptance by the molecule. The values of the *E*_LUMO_ listed in Table 2 for the studied complexes are in the order MnLL′ > CoLL′ > ZnLL′ > CuLL′, which implies that the CuLL′ has the highest tendency to accept electrons from the appropriate occupied orbitals of an electron-donating species. The values of some thermodynamic parameters such as BE, Δ*H*, Δ*S*, and Δ*G* for the studied metal complexes are reported in Table 2. The negative values of the BE in Table 2 indicate that the products of the reaction depicted by the equation shown in Figure 3 are more stable than the reactants. In other words, high amount of energy is required to split the metal complexes into their constituent metal ions and ligands, which is an indication of favourable formation of the complexes. The magnitudes of the BE values show that the CuLL′ complex requires the highest amount of energy to break it into the constituent ligands and Cu^2+^ ion, making it the most stable complex. The negative values of Δ*H* imply that the reactions leading to the formation of the metal complexes are exothermic. The results in Table 2 also show that the Δ*G* values for the formation of the metal complexes are negative, which imply that the formation of the metal complexes is a spontaneous reaction. The order of spontaneity for the formation of the six-coordinate complexes is CuLL′ > CoLL′ > MnLL′, which suggests that the CuLL′ is the most stable complex. This is in agreement with the relative strength of the metal–ligand covalent bonds deduced from the spin density distribution analyses and the strength of the H–bonds involved in the optimized structures of the metal complexes. The overall trend of the stability of the metal complexes observed in the present study, that is, Mn < Co < Cu > Zn, is in good agreement with the observation of Irving and Williams on the complexes of some first-row transition metals regardless of the nature of the ligand involved and the number of coordinated ligands [74]. Similar trend was also observed in the work of Luther et al. on the experimental stability constants of metal (bi)sulfide complexes of some first-row transition metals [75].

## 5. Biological Studies

### 5.1. Antimicrobial Screening

The assay was carried out on the metal(II) complexes using agar diffusion technique [76]. The surface of the agar in a Petri dish was uniformly inoculated with 0.3 mL of 18 hour-old test bacteria/fungus culture. Using a sterile cork borer, 6 mm wells were bored into agar. Then 0.06 mL of 10 mg/mL concentration of each metal complex in DMSO was introduced into the wells and the plates were allowed to stand on bench for 30 min before incubation at 37°C for 24 h. After this, the inhibitory zones (in mm) were taken as a measure of antibacterial and antifungal activity and presented in Table 3. The experiments were conducted in duplicate and streptomycin and fluconazole were used as the reference drug for the test bacteria and fungi, respectively.

**Figure 6:**
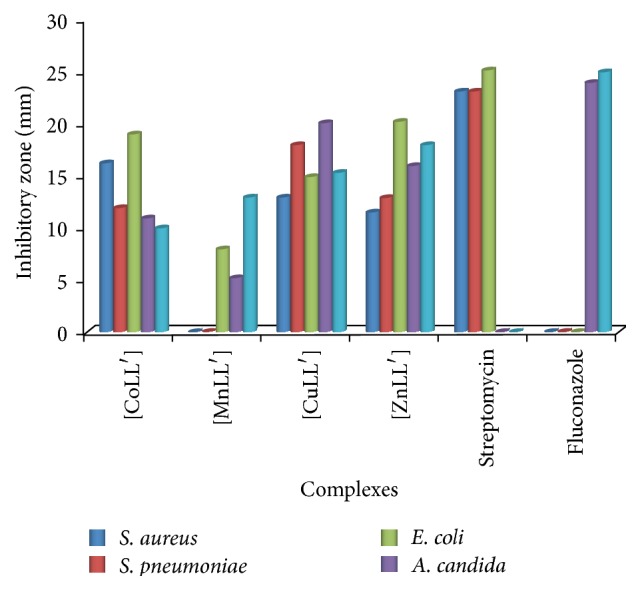
Histogram presentation of antimicrobial activity of the mixed ligand complexes.

The test compounds were screened against two Gram positive bacteria (*S. aureus* and* S. pneumoniae*), one Gram negative bacterium (*E. coli*), and two fungi organisms (*A. niger* and* A. candida*). The results presented in Table 3 and Figure 6 show that the test compounds have a low to high antimicrobial activity against the microbes. The CuLL′ and ZnLL′ complexes exhibited 83.7% activity of streptomycin against* E. coli*. The ZnLL′ exhibited 72% and 80.5% activity compared to fluconazole against* A. niger* and* A. candida*, respectively. Generally, the ZnLL′ complex showed the overall best antimicrobial character among the test compounds.

## 6. Conclusions

New heteroleptic complexes of Zn(II), Cu(II), Mn(II), and Co(II) derived from the mixed ligands of* N*-methyl-*N*-phenyldithiocarbamate and benzoylacetone have been synthesized and characterized by various physicochemical methods. The Zn complex displayed a four-coordinate geometry, while the remaining complexes (Cu(II), Co(II), and Mn(II)) adopted six-coordinate systems each with two molecules of water as additional ligands. DFT calculations generated some electronic and thermodynamic parameters, which indicates that the Zn(II) complex has the highest tendency to donate its most energetic electron, while the Cu(II) complex has the highest tendency to accept electrons from the appropriate occupied orbitals of an electron-donating species. The overall trend of the stability of the metal complexes observed in the present study is Mn < Co < Cu > Zn. The results of the antimicrobial activity studies show that the zinc complex is more effective compared to the other complexes.

## Figures and Tables

**Table 1:** ^*∗*^Atomic percentage contributions to the spin density delocalization derived from the Mulliken atomic spin density values.

Complex	M	O_sp^2^_	O_sp^3^_	O (OH_2_)	N	S_sp^2^_	S_sp^3^_	C (–CS_2_–)	C_alp/ar_
MnLL′	99.81	0.00080	0.03053	0.00929	0.00488	0.00388	0.00254	0.13575	0.00351^ar^
CoLL′	96.80	0.00013	0.52223	0.04193	0.00151	0.44764	2.09192	0.06274	0.00276^alp^
CuLL′	33.07	0.00149	23.72	0.98211	0.01081	39.84	0.00118	0.03101	0.11547^alp^

^*∗*^Percentage contributions by atom were calculated as the ratio of square of Mulliken spin density for an atom and the sum of square of Mulliken spin density for all atoms in the molecule. M = Mn, Co, or Cu; C (–CS_2_–) is the carbon atom of the dithiocarbamate group. Highest contributions from the carbon atoms farther from the coordination sites were found on an aromatic/aliphatic carbon of the benzoylacetone ligand: ar = highest contribution from an aromatic carbon; alp = highest contribution from an aliphatic.

**Table 2:** ^a^Some electronic and thermodynamic parameters of ZnLL′, MnLL′, CoLL′, or CuLL′.

Parameters	ZnLL′	MnLL′	CoLL′	CuLL′
*E*_HOMO_ (eV)	−5.62	−5.01	−5.43	−5.59
*E*_LUMO_ (eV)	−2.40	−2.12	−2.20	−2.93
*μ* (Debye)	4.39	0.81	1.58	3.57
BE (kcal/mol)	−394.16	−299.11	−381.26	−418.24
Δ*H* (kcal/mol)	−394.48	−301.71	−383.49	−419.98
Δ*S* (cal/mol)	−68.06	−147.66	−144.23	−134.10
Δ*G* (kcal/mol)	−374.18	−257.68	−340.49	−380.00

^a^All thermodynamic parameters are corrected for zero-point and thermal energies at 298 K. Thermodynamic data for monatomic M^2+^ (M = Zn, Mn, Co, and Cu) ions were obtained from B3LYP/LANL2DZ theory.

**Table 3:** Summary of antimicrobial screening of the mixed ligand complexes.

Name	*S*. *aureus*	*S*. *pneumoniae*	*E*. *coli*	*A*. *candida*	*Aspergillus niger*
[CoLL′]	16.3 ± 0	12.0 ± 0.1	19.0 ± 0.2	11.0 ± 0.1	10.0 ± 0.03
[MnLL′]	R	R	8.0 ± 0.4	5.2 ± 0.7	13.0 ± 0.7
[CuLL′]	13.0 ± 0.01	18.0 ± 0.1	15.0 ± 0.3	20.1 ± 0.02	15.3 ± 0.01
[ZnLL′]	11.5 ± 0.03	13.0 ± 1.4	20.2 ± 0.12	16.0 ± 0	18.0 ± 0.1
Streptomycin	23.2 ± 0.1	23.2 ± 0.03	25.1 ± 0	—	—
Fluconazole	—	—	—	24.0 ± 0.1	25.0 ± 0
DMSO	R	R	R	R	R
